# Unclassifiable renal carcinoma with medullary phenotype and SMARCB1 deficiency: case report

**DOI:** 10.3389/fruro.2025.1582675

**Published:** 2025-06-06

**Authors:** Samya H. Mehanna, Thiago Gabriel Ronkoski, Cassiano Machado, Lucas S. Wolff, Alexandre C. Cavalli, Thiago Hota, Fernando C. Koleski

**Affiliations:** ^1^ Department of Pathology, Nossa Senhora das Graças Hospital, CURITIBA-PR, Curitiba, Brazil; ^2^ Faculdade Evangélica do Paraná, Curitiba-PR, Brazil; ^3^ Department of Urology, Nossa Senhora das Graças Hospital, Curitiba-PR, Brazil

**Keywords:** renal cell carcinoma, renal medullary carcinoma, INI1 deficiency, phenotype, hemoglobinopathy

## Abstract

**Background:**

Renal medullary carcinoma (RMC) is an aggressive tumor representing less than 0.5% of renal cell carcinomas (RCC), and it is considered rare. When it occurs, patients typically have sickle cell trait, sickle cell disease, or an associated hemoglobinopathy, which is a necessary characteristic for diagnosis. Additionally, RMC is characterized by the inactivation of alterations in the SMARCB1 (INI1) tumor suppressor gene, resulting in the loss of INI1 immunohistochemical expression. However, there are tumors reported in the literature with the same morphological and phenotypic characteristics as RMC but without hemoglobinopathy, referred to as “unclassified RCC with medullary phenotype.”

**Case report:**

We present the 13th case of unclassified renal cell carcinoma with a medullary phenotype in a 20-year-old woman. The patient was admitted with complaints of macroscopic hematuria, with no significant findings on physical examination. Diagnostic investigation included a computed tomography urogram, which revealed a hypovascular oval image with central cystic/necrotic areas in the middle third of the right kidney, measuring 32 mm, suggesting a possible diagnosis of an infected renal cyst. Subsequent magnetic resonance imaging showed findings consistent with an atypical presentation of primary neoplasia in the differential diagnosis, prompting a renal biopsy for case definition. Histopathological analysis revealed a high-grade infiltrative epithelioid neoplasm. Immunohistochemistry showed positivity for PAX8 and loss of INI-1 expression. No hemoglobinopathies were identified in the patient, in this context, the neoplasm is appropriately classified as unclassified renal cell carcinoma (RCC) with medullary phenotype and SMARCB1 deficiency. The instituted therapy consisted of right radical nephrectomy with retroperitoneal lymphadenectomy, with nodal metastases detected.

**Conclusion:**

Given the rarity of unclassified RCC with a medullary phenotype, continuous documentation and analysis of individual cases not associated with sickle cell trait are crucial to understanding its behavior, prognosis, and potential therapeutic approaches, considering its aggressiveness and high metastatic potential.

## Introduction

Renal medullary carcinoma (RMC) is an aggressive tumor accounting for less than 0.5% of renal cell carcinomas (RCC) (1), it is a rare entity that primarily occurs in patients in their third decade of life, and in more than 95% of cases, patients exhibit sickle cell trait, sickle cell disease, and associated hemoglobinopathies (2).

RMC is characterized by the inactivation of alterations in the SMARCB1/INI1 tumor suppressor gene, resulting in the loss of INI1 immunohistochemical expression (3). The presence of hemoglobinopathy, determined by clinical history, hemoglobin electrophoresis, or the identification of sickle cells in tissue sections, is often considered a general prerequisite for this diagnosis.

Recently, extremely atypical and uncommon tumors that share morphological and phenotypic characteristics with RMC, but occur in patients without hemoglobinopathy, have been reported and temporarily termed “unclassified RCC with medullary phenotype and SMARCB1 deficiency” (4).

Given these notions, the primary benefit of this work is to contribute to the existing knowledge on the topic by presenting the thirteenth case of metastatic RCC with SMARCB1 deficiency described in the literature. This case involves a 20-year-old female patient without hematological abnormalities, detailing clinical, radiological, and histopathological aspects.

## Case report

A 20-year-old Caucasian female patient, a student, presented with symptoms of lower urinary tract irritation and weight loss (associated with dietary changes). She denies any history of comorbidities, previous surgeries, or continuous medication use. She sought medical attention for painless macroscopic hematuria with a 20-day evolution and no other reported complaints. On physical examination, she had a flat, flaccid, non-tender abdomen with no palpable visceromegalies. Empirical treatment for urinary tract infection was initially prescribed, and laboratory and imaging tests were requested.

During the analysis by total abdominal ultrasound, the presence of a renal nodule on the right side was detected. It was decided to proceed with the investigation using a computed tomography urography, which revealed a hypovascular oval image with central areas of cystic/necrotic appearance in the middle third of the right kidney, measuring 32 mm, in contact with the renal pelvis, suggesting a possible diagnosis of an infected renal cyst. Additionally, further investigation with magnetic resonance imaging was performed, maintaining the same findings suggestive of a complicated cyst or caliceal diverticulum with inflammatory changes in the renal parenchyma.

**Figure 1:**
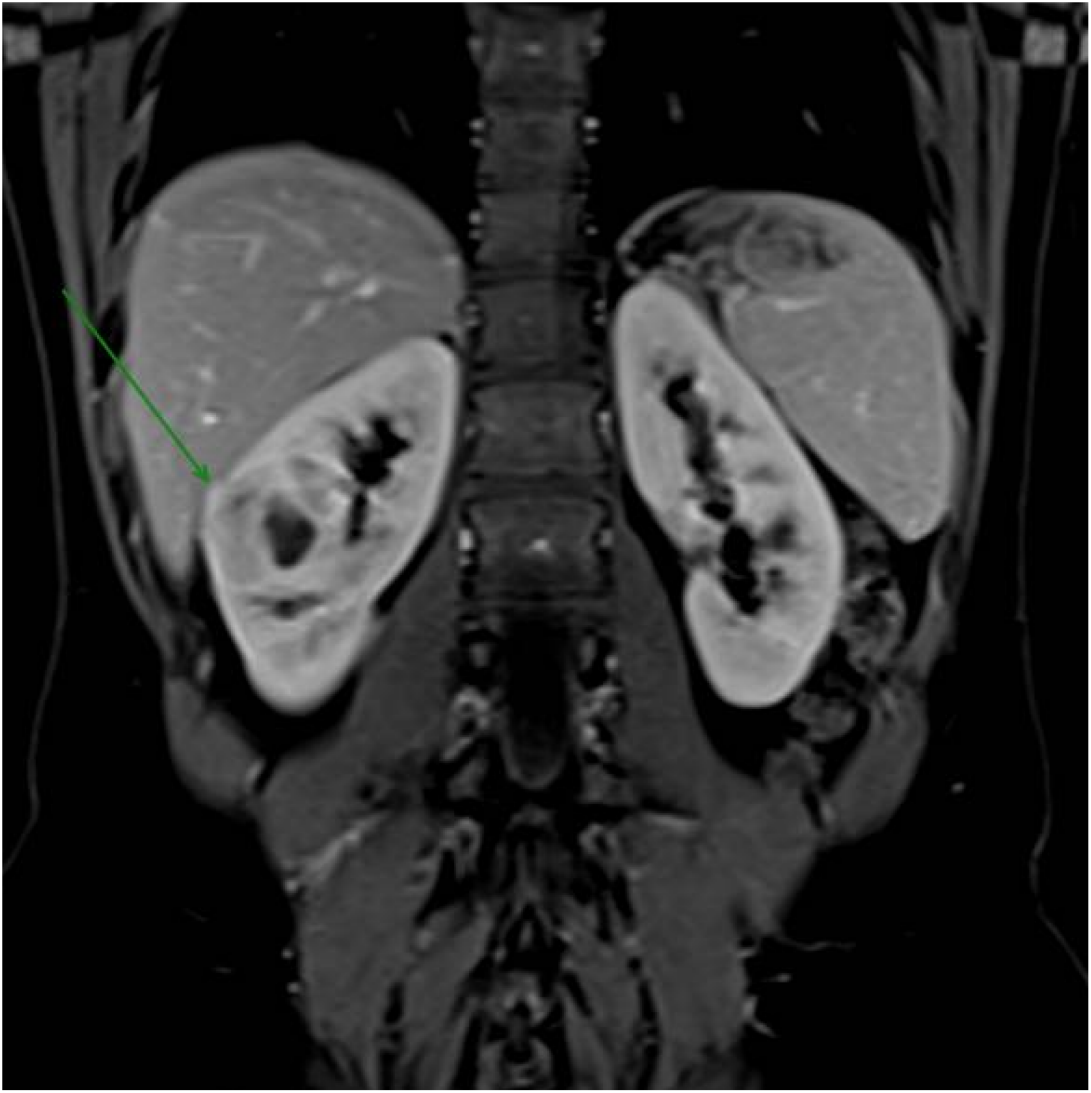
Magnetic resonance imaging revealed a right renal mass measuring approximately 44 mm in its largest dimension, increasing from 32 mm previously, characterizing a predominantly endophytic mass of indeterminate nature.

In follow-up for early evolutionary control, a new magnetic resonance imaging was performed, which observed an increase in the hypovascular solid component, with restricted water diffusion around the cyst, with a hemorrhagic component in the anterolateral aspect of the middle third of kidney; an increase in the right renal mass (from 32 mm to approximately 44 mm in the largest axis), characterizing a predominantly endophytic mass of indeterminate nature. However, due to the behavior over the interval since the previous examination, the possibility of an atypical presentation of primary neoplasia was considered in the differential diagnosis, without signs of macroscopic invasion of the collecting system or adjacent vascular structures (R.E.N.A.L. Score: 2 + 2 + 3+a+3 = 10a) (**Figure 1**).

Renal biopsy guided by imaging was requested for diagnostic elucidation. Microscopically, it revealed the presence of a high-grade epithelioid infiltrative neoplasm composed of round and ovoid cells with marked atypia, arranged in solid and tubular patterns, associated with intense desmoplastic stromal reaction. Immunohistochemical study showed positivity for PAX8 and deficiency of INI-1 (SMARCB1) expression, consistent with Renal Carcinoma with INI-1 deficiency.

**Figure 2:**
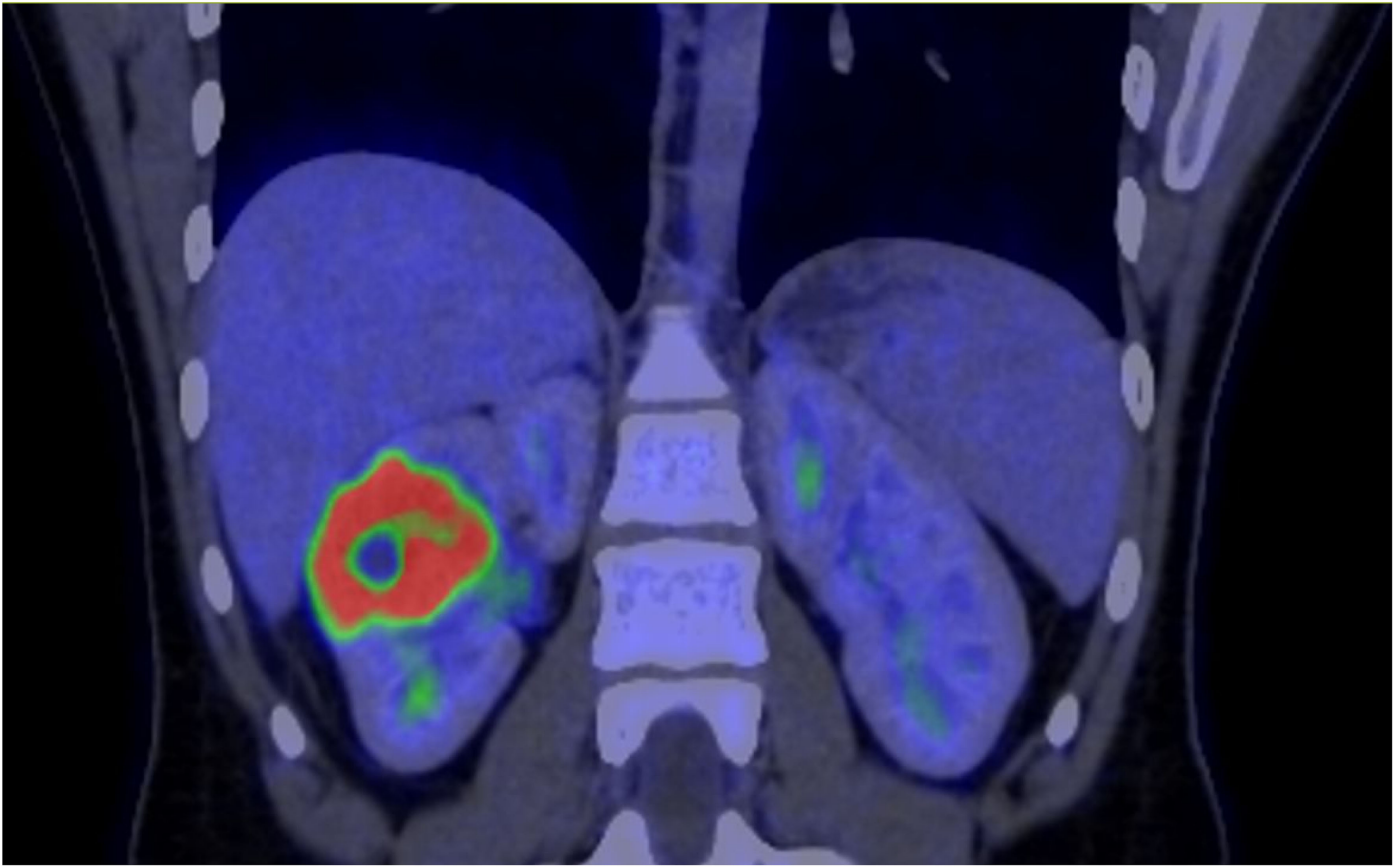
PET-CT showed evidence of a renal lesion with marked hypermetabolism and a central necrotic area in the anterolateral cortical region of the middle third of the right kidney.

**Figure 3:**
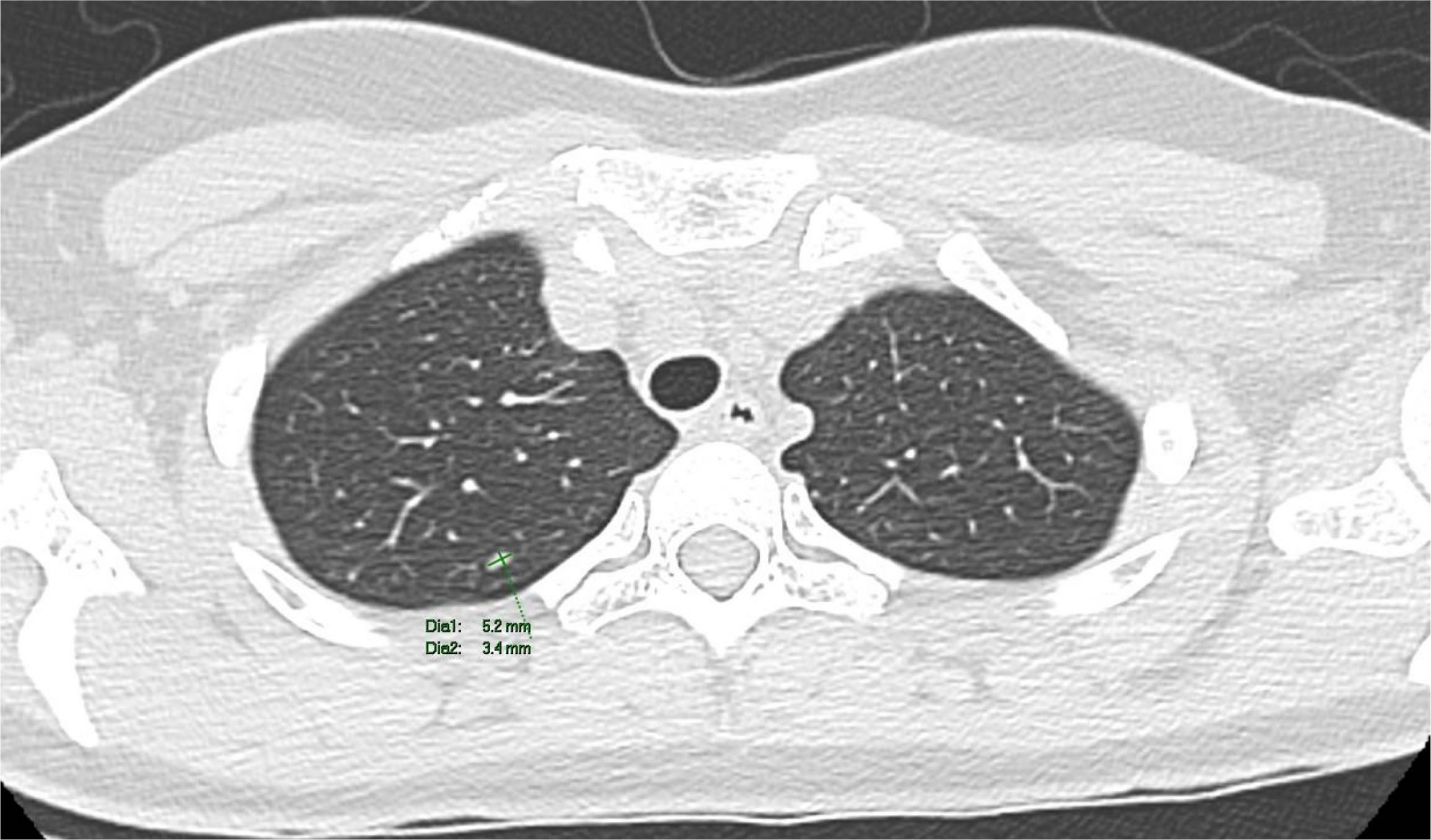
A computed tomography scan revealed non-calcified nodules in the upper lobe of the right lung.

**Figure 4:**
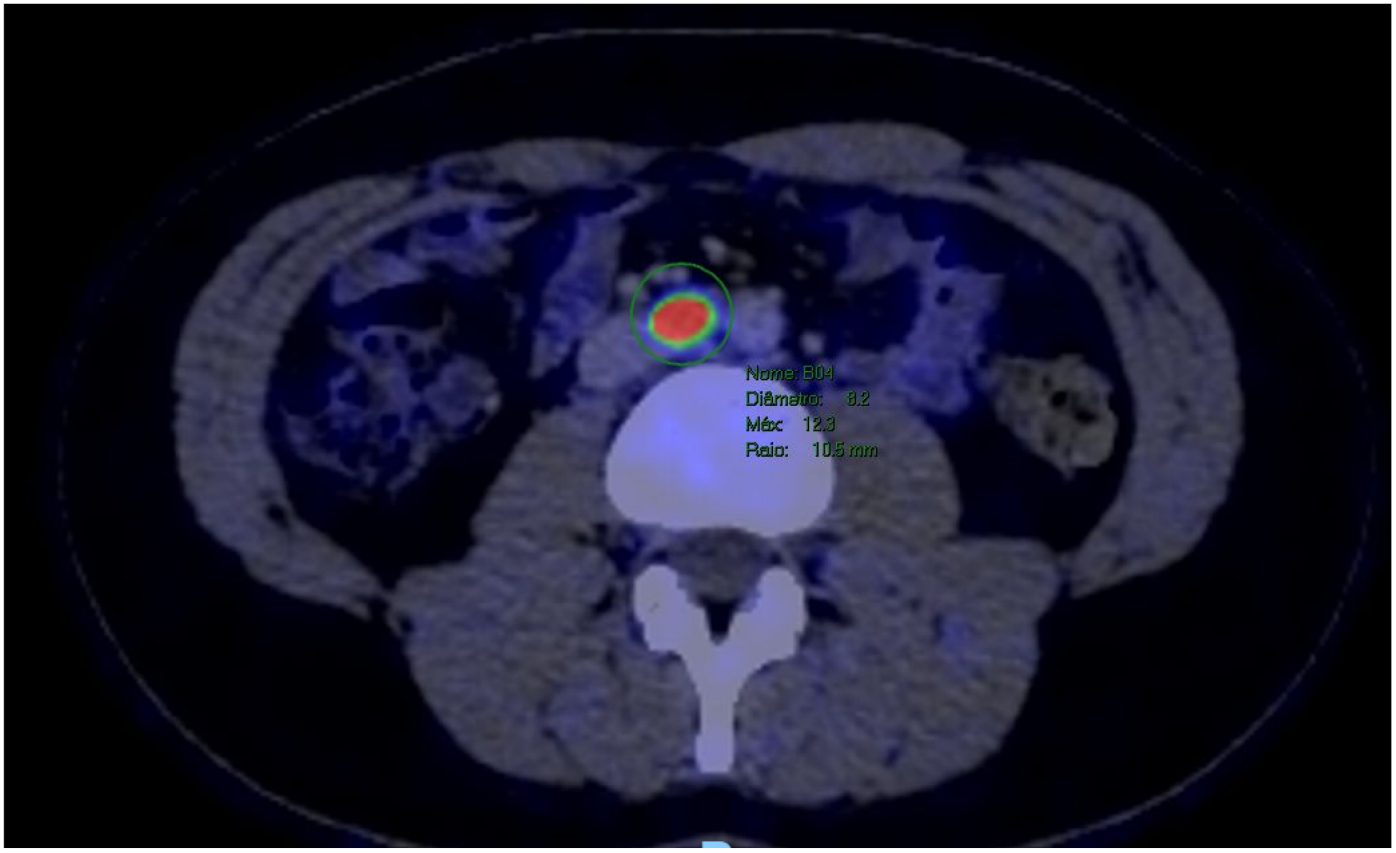
PET-CT showed a lymph node with marked hypermetabolism in the interaortocaval region.

The patient underwent a PET-CT scan for staging, revealing an expansive/infiltrative lesion with marked hypermetabolism and central necrotic area located in the anterolateral cortical area of the middle third of the kidney. This lesion increased in size compared to the previous study, measuring approximately 55 x 38 x 36 mm in its largest dimensions, consistent with primary neoplasia. There was also enlargement of lymph nodes showing marked hypermetabolism in the paracaval region adjacent to the renal hilum and in the interaortocaval region, suspicious for neoplastic spread. Additionally, a lymph node with slightly heterogeneous enhancement on contrast and marked hypermetabolism in the left pulmonary hilum was suspicious for metastasis. There were also scattered small non-calcified nodules throughout the lungs, the largest of which showed faint uptake of the radiotracer in the upper lobe of the right lung, of indeterminate nature, potentially representing malignant lesions. (**Figures 2**–**4**).

**Figure 5:**
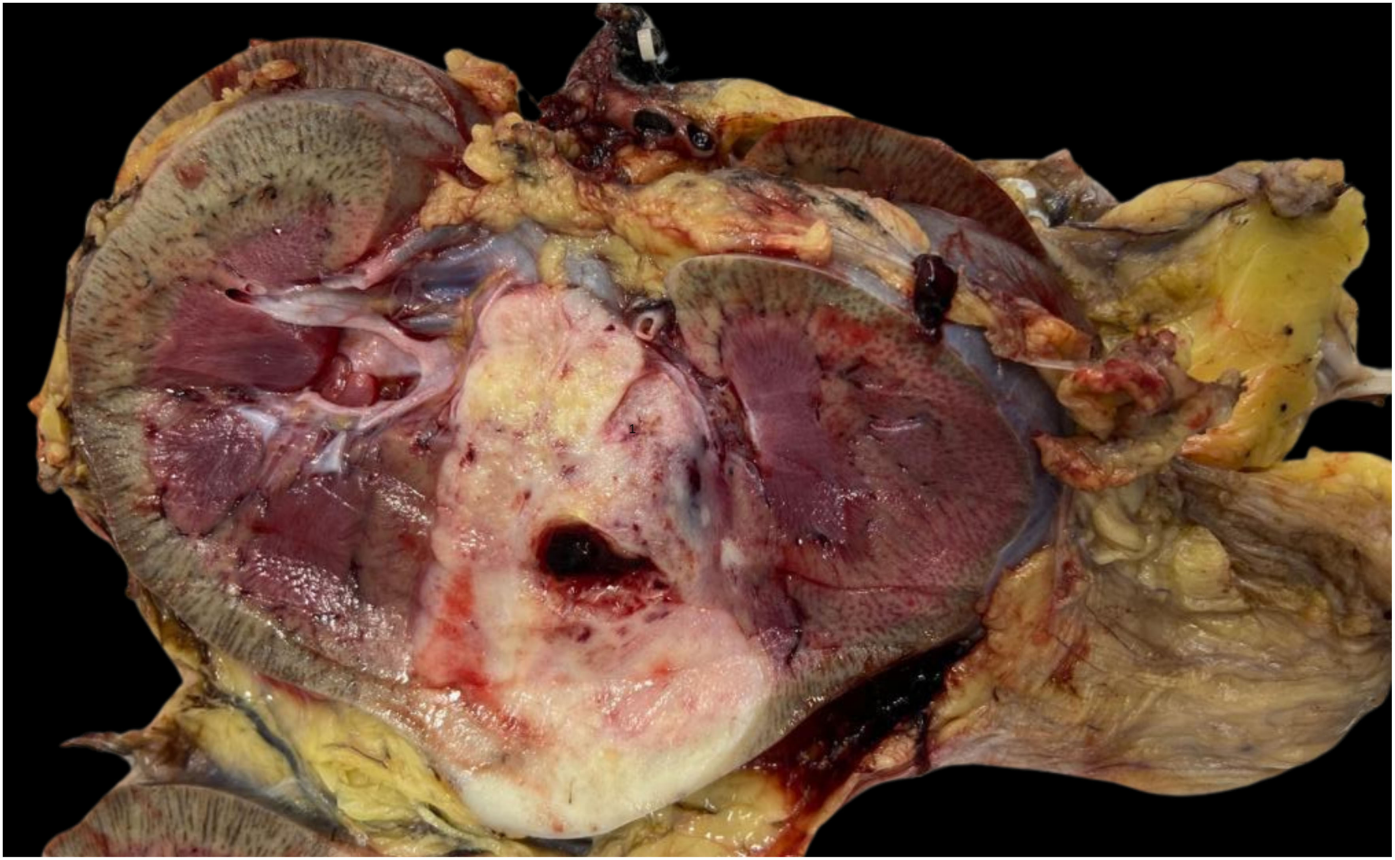
Macroscopic image of renal tumor measuring 5.5 x 4.2 cm in its largest dimensions, located in the middle third at the cortical/medullary interface.

The therapeutic approach involved performing a radical nephrectomy with retroperitoneal lymph node dissection. The surgical specimen contained a solid-cystic nodular lesion, brownish-white in color, firm and elastic in consistency, measuring 5.5 x 4.2 cm in its largest dimensions. It was located in the middle third at the cortical/medullary interface, extending into the perirenal fat and renal sinus (**Figure 5**).

**Figure 6:**
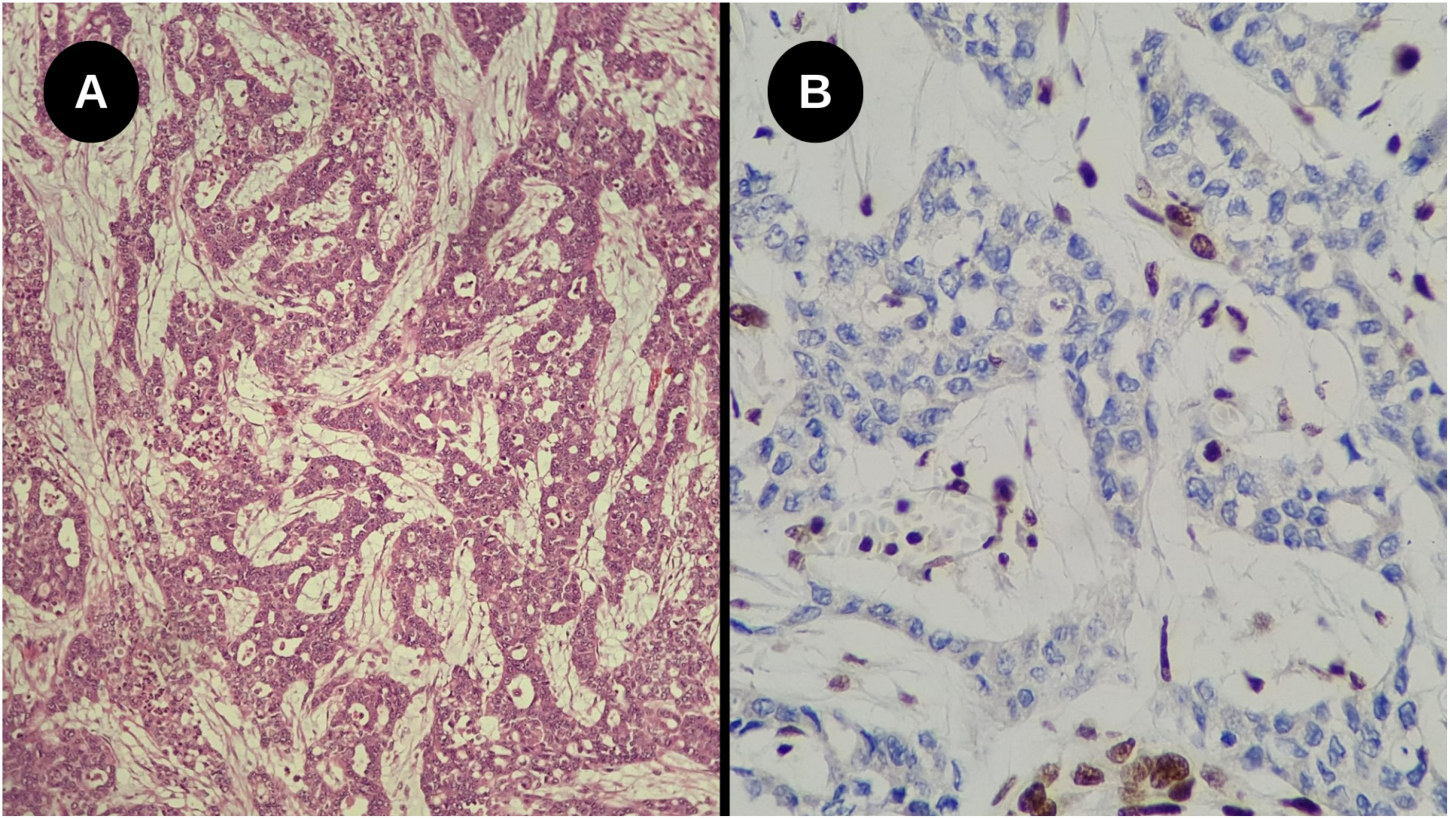
**(A)** High-grade infiltrative glandular neoplasm with reticular and cribriform growth pattern, associated with a marked desmoplastic stromal response. No rhabdoid components were identified (Hematoxylin-eosin staining, Optical microscopy, magnification 200x); **(B)** Loss of nuclear INI-1 expression in tumor cells, with positive internal control in adjacent cells (Immunohistochemistry, Optical microscopy, magnification 40x).

On histopathological analysis, it is characterized as high-grade renal carcinoma, consisting of neoplastic growth with an infiltrative adenocarcinoma-like pattern, forming nests, microcysts, and tubules surrounded by myxoid desmoplastic reaction (**Figure 6A**), involvement of the renal sinus and signs of angiolymphatic invasion. No rhabdoid or sarcomatoid components were observed. Surgical margins were clear, but there were metastases in four retroperitoneal lymph nodes and one in the region of the renal artery, all showing extranodal extension. The pathological staging (TNM) was pT3a pN1. Subsequent immunohistochemical study of the tumor confirmed the deficiency of INI-1 with negativity of the antibody tested (**Figure 6B**).

The patient showed good postoperative progress and was discharged on the second day. Following diagnosis, it is crucial to clinically investigate sickle cell anemia, sickle cell trait, and other hemoglobinopathies to correlate with the histopathological diagnosis of Renal Medullary Carcinoma (RMC). The patient underwent screening for hemoglobinopathies, which yielded negative results. Thus, due to the absence of hematological alterations and distinct epidemiological profile, the neoplasm is appropriately classified as unclassified renal cell carcinoma (RCC) with medullary phenotype and SMARCB1 deficiency. No additional molecular tests were performed.

Furthermore, treatment with cisplatin and gemzar was administered. In a subsequent PET-CT scan performed 6 months after the surgical procedure, there was reduction in the lymph nodes in the pulmonary hilum, stability in tiny pulmonary nodules, and no significant changes related to the right nephrectomy and retroperitoneal lymph node dissection. No new lesions suspicious for recurrence were identified in the surgical bed or elsewhere. After initial follow-up, the patient continued treatment at another external service. The patient deceased within six months.

## Discussion

Renal medullary carcinoma (RMC) is a rare neoplasm that comprises less than 0.5% of renal carcinomas (5) which generally affects young African Americans in their second or third decade of life with hemoglobinopathies, especially those with sickle cell trait (6, 7), for this reason, it is also known as the “seventh sickle cell nephropathy” (8). The occurrence of RMC without the presence of hemoglobinopathy is even rarer (6), and this article reports the 13th case of RMC without associated hemoglobinopathy, classified according to the 4th edition of the WHO Classification of Tumors of the Urinary System and Male Genital Organs as unclassified renal cell carcinoma (RCC) with medullary phenotype and SMARCB1(INI1) deficiency (9). All cases reported in the literature are presented below in **Table 1**, along with their most significant characteristics (4, 10–16).

**Table 1:** Clinica, histopathological and molecular characteristics of the 12 cases of Unclassified renal cell carcinoma (RCC) with medullary phenotype and SMARCB1(INI1) deficiency and no hemoglobinopathy described in the literature.

CASE	1	2	3	4	5	6	7	8	9	10	11	12
SEX	Male	Male	Female	Male	Male	Male	Male	Male	Male	Female	Male	Male
AGE	62	76	15	63	71	33	39	71	58	24	30	42
NAT	CA	CA	HIS	EAS	NE	NE	CA	CA	CA	CA	EAS	NE
SIZE* (cm)	13.0	6.3	8.7	4.3	3.3	5.8	19.0	6.5	3.4	5.5	4.5	5.5
RL	RK-UP	RK-MP	RK-NE	LK-UP	RK-UP	LK-LP	RK-NE	LK-NE	LK-NE	LK-NE	RK-NE	LK-NE
VI	+	–	–	–	–	–	–	–	–	–	–	–
FI	+	+	–	+	–	–	–	–	–	–	–	–
LNM	+	–	+	+	+	+	+	+	+	–	+	+
DM	+	+	+	–	–	+	–	+	+	–	+	–
INI1	–	–	–	–	–	–	–	–	–	–	–	–
PAX8	+	+	+	+	+	+	+	+	+	+	+	+

NAT, Naturalness; CA, Caucasian; HIS, Hispanic; EAS, Eastern; NE, Not specified; RL, Renal location; VI, Vascular invasion; FI, Fat invasion; LNM, Lymph node metastasis; DM, Distant metastasis; UP, Upper pole; MP, Middle pole; LP, Lower pole; LK, Left kidney; RK, Right kidney.

*Largest diameter.

### Epidemiology and clinic

The population most affected by RMC is young males with an average age of 26 years of African or Mediterranean descent (7), who seek medical services reporting hematuria and/or flank pain (6), this creates a significant problem due to the nonspecificity of these symptoms, delaying the probability of a diagnostic hypothesis for the disease. In addition to these symptoms, Berman (17) highlights other clinical features such as papillary necrosis, nephritic syndrome, renal infarction, inability to concentrate urine, and pyelonephritis. Blas et al. (7) also mention respiratory difficulty, palpable mass, cough, and fever.

The presentation of these systemic symptoms generally depends on the extent of the disease. It is important to note that RMC is highly aggressive, with metastases being extremely common early in the course of the disease (6, 7), the most affected sites are lymph nodes, liver, lungs, bones, and adrenal glands (18), resulting in low survival rates.

### Pathogenesis

It is believed that RMC is triggered by chronic hypoxia due to inadequate blood supply caused by sickle cells (5), additionally, there is an important association with the SMARCB1 gene (SWI/SNF-related, matrix-associated, actin-dependent regulator of chromatin, subfamily B, member 1), which acts as a tumor suppressor on chromosome 22 at position 11.23 (22q11.23) (19, 20), considering its loss in the SWI/SNF complex, which mediates chromatin remodeling and modulates transcriptional activity in various neoplasms, it suggests its suppressor function (5). The most modern theory suggests that regional ischemia caused by sickling, combined with extreme hypoxia and hypertonicity of the renal medulla, triggers mechanisms that lead to DNA restructuring, including deletions and translocations in SMARCB1, resulting in its inactivation. It is important to note that hematuria occurs more frequently in the left kidney, but as shown, RMC predominantly affects the right kidney. This is explained by anatomy, where the right renal artery, being longer than the left, results in reduced blood flow to the kidney (21).

### Macroscopy

The morphology is characterized by an ill-defined, poorly circumscribed mass with a firm or rubbery consistency and a surface of varying coloration, ranging from bronze to gray, occupying a large part of the renal medulla. It varies in size from 2–18 cm with an average diameter of 7.4 cm and predominantly affects the right kidney three times more often. Infiltrations into the renal pelvis, perirenal adipose tissue, and renal sinus are common, along with changes such as cysts, hemorrhage, and necrosis (1, 8, 22).

### Histopathology and immunochemistry

Microscopically, a reticular or cribiform pattern can be observed with marked desmoplastic stromal response, necrosis, and inflammatory infiltrate, especially neutrophilic. Other patterns found include adenoid cystic, sarcomatoid, and microcystic (6, 23). Many cases exhibit focal rhabdoid characteristics, characterized by large eccentrically positioned nuclei, prominent nucleoli, eosinophilic cytoplasm, and atypical mitoses (24, 25). Among the differential diagnoses that should be considered are: collecting duct carcinoma, malignant rhabdoid tumor, urothelial carcinoma, renal cell carcinoma with vinculin-ALK translocation-anaplastic lymphoma kinase (VCL-ALK), among others (6, 7).

Due to the rarity of this disease, immunohistochemistry has been used as an auxiliary method for diagnosis. RMC may show positivity for low molecular weight cytokeratin 8,18 (CAM 5.2), pancytokeratin (AE1/AE3), vimentin, PAX-8, epithelial membrane antigen (EMA), among other markers. However, the hallmark of the disease is the complete loss of integrase interactor 1 of the tumor suppressor gene (INI1), also known as SMARCB1, which is critical for diagnosis (6). The significant importance of not detecting this gene is that in all renal cell carcinomas (RCC) or urothelial cell carcinomas, there is expression of INI1 (26). When histological and immunohistochemical findings compatible with RMC are found, but the patient’s sickle cell trait status is unknown, hemoglobin electrophoresis can be performed to detect hemoglobin S. If hemoglobin S is not found and no other hemoglobinopathies are present, the tumor is classified as unclassified renal cell carcinoma (RCC) with medullary phenotype and SMARCB1 deficiency (6).

In addition to immunohistochemistry for INI1 expression loss, other molecular tests can be used to diagnose renal medullary carcinoma with SMARCB1 deficiency. Fluorescence *in situ* hybridization (FISH) analysis is a technique that can be employed to assess the status of the SMARCB1 locus. Studies have shown that in cases of renal medullary carcinoma, there may be hemizygous loss and translocation of SMARCB1, homozygous loss, or, in some cases, no structural or copy number alteration despite the protein loss. Furthermore, next-generation sequencing can identify pathogenic somatic mutations in SMARCB1, even in cases that do not show detectable alterations by FISH (27).

### Differential diagnosis

As mentioned, the main differential diagnoses include collecting duct carcinoma (CDC), urothelial carcinoma, malignant rhabdoid tumor of the kidney, and renal cell carcinoma with VCL-ALK translocation. CDC histologically may resemble unclassified renal cell carcinoma (RCC) with medullary phenotype and SMARCB1 deficiency but differs by presenting a tubular or tubulopapillary pattern, whereas RMC exhibits a reticular pattern (28), regarding age range, CDC generally affects individuals over 55 years old, whereas RMC rarely occurs in patients over 40 years old (29), finally, from an immunohistochemical standpoint, the loss of INI1 expression in RMC is highlighted, which is an important method for distinguishing these neoplasms (30). The rhabdoid tumor was not included in the differential due to incompatible histopathological characteristics, in addition to the patient’s age, which differs from the case report for this neoplasia.

The main features distinguishing collecting duct carcinoma (CDC) from unclassified renal cell carcinoma (RCC) with medullary phenotype (28) are summarized in **Table 2**.

**Table 2:** Clinical, histopathological, and immunohistochemical differences between Collecting Duct Carcinoma (CDC) and unclassified Renal cell carcinoma (RCC) with medullary phenotype.

Diagnosis	Collecting duct carcinoma (CDC)	Unclassified Renal cell carcinoma (RCC) with medullary phenotype
Average age (years)	65	48
Group affected	Caucasian	Caucasian
Tumor Size (cm)	6,3	7,1
Tumor location (%)	Medullary (41%), Medullary/cortex (44%)	Medullary (100%)
Histopathology	High-grade adenocarcinoma, irregular tubular, tubulopapillary or glandular architecture, with infiltrative growth and stromal desmoplasia	High-grade adenocarcinoma, reticular or cribriform pattern with a marked desmoplastic stromal response and a robust inflammatory infiltrate
Immunohistochemistry for SMARCB1/INI1	Expression preserved	Lost expression
Percentage of lymph node metastasis (%)	48%	83%
Percentage of distant metastasis (%)	73%	58%

Similarly, urothelial carcinoma of the renal pelvis shows expression of INI1/SMARCB1, maybe positive for PAX-8, and tends to occur in older patients (18, 31). Regarding malignant rhabdoid tumor of the kidney, its morphology resembles RMC, and immunohistochemically, this neoplasm also shows loss of INI1 expression, but it occurs in patients younger than 3 years old (5, 32, 33). As for renal cell carcinoma VCL-ALK, it also occurs in patients with sickle cell trait and histological findings compatible with RMC, however, there is no deficiency of SMARCB1 expression (24, 25).

### Treatment

Given its high aggressiveness, unclassified renal cell carcinoma (RCC) with medullary phenotype and SMARCB1 deficiency presents a poor prognosis, with a survival of four months for patients with metastases and seventeen months for those without metastases (34). Generally, there is resistance to many chemotherapy agents, with the most commonly used therapies being platinum-paclitaxel-gemcitabine therapy, topoisomerase inhibitor therapy, and methotrexate-vinblastine-doxorubicin-cisplatin therapy, which increase survival by 12, 7, and 4 months, respectively (6). Radical nephrectomy is recommended at the time of diagnosis for localized disease, potentially increasing survival (from 3 to 6 months) and improving symptom management. However, there is still no substantial evidence of significant improvement when metastases have already occurred (33).

With significant therapeutic potential in the literature, Batra’s study (35) highlights anti-angiogenic therapy, reporting a case where bevacizumab and temozolomide were used for 6 months, leading to remission for 42 months. Additionally, other therapies such as sunitinib, PD-1 inhibitor nivolumab, and EZH2 inhibitors are mentioned. In summary, there is no definitive recommended treatment, and approaches are often adopted from treatments used for other malignancies, with poor responses to treatment and a dismal prognosis. The most common approaches include systemic therapies and nephrectomy (7).

### Future perspectives

As can be observed, current therapies remain very limited and generally offer low survival rates. Consequently, studies are underway focusing on targeted therapies, including proteasome inhibitors (ixazomib), EZH2 inhibitors, and mTOR inhibitors (everolimus). However, these approaches have not yet been standardized (36). Therefore, there is a clear need for a deeper understanding of the molecular characteristics of this neoplasm in order to develop more effective therapies that could improve patient survival or even achieve a cure.

## Conclusion

The diagnosis of renal carcinoma with SMARCB1 deficiency remains challenging due to the rarity of this neoplasm and the nonspecific initial symptoms, which may mimic lower urinary tract diseases. Thus, a high index of clinical suspicion is crucial for early diagnosis, potentially improving patient survival rates given the aggressive behavior and high metastatic potential of this tumor. Immunohistochemical analysis is essential for diagnostic confirmation, and treatment represents a major ongoing challenge due to the limited therapeutic options currently available.

This case highlights the importance for pathologists and oncologists to consider this rare entity in differential diagnoses, particularly in atypical presentations. Additionally, future research efforts and the establishment of prospective registries are critical to developing standardized treatment protocols and identifying novel biomarkers that may enable earlier detection and targeted therapies.

## Data Availability

The original contributions presented in the study are included in the article/supplementary material. Further inquiries can be directed to the corresponding author/s.
